# The Calhoun Experiment Study by Means of Agent-Based Modeling

**DOI:** 10.3390/e28020169

**Published:** 2026-02-01

**Authors:** Tomasz M. Gwizdałła, Jakub Duś

**Affiliations:** Faculty of Physics and Applied Informatics, University of Łódź, Pomorska 149/153, 90-236 Łódź, Poland; jakub.dus@edu.uni.lodz.pl

**Keywords:** behavioral sink, Calhoun’s experiment, social phenomenon modeling

## Abstract

More than 50 years ago John D. Calhoun conducted a series of experiments devoted to studying the social behavior of mice. The longest of them lasted more than four years and led to the creation of the concept of the so-called “behavioral sink”. The population of mice, which had all possible resources at their disposal but were located in a limited room, disintegrated after an initial phase of strong development. In our paper we are going to reproduce the effects of this experiment. The crucial problem in every simulation approach is to determine the set of the most important parameters which influence the global as well as local effects of the simulated process. In the studied case we have the problem that a lot of important information is missing. The author of the original work focused rather on social mechanisms, often omitting key numerical data related to the course of the experiment. In this paper we try to propose a certain set of parameters. By using them we can reproduce Calhoun’s results qualitatively and, with some deviation, quantitatively.

## 1. Introduction

In the 1960s and 1970s, John B. Calhoun performed a series of experiments to study the behavior of mice in a closed environment and the social effects of a significant reduction in a population’s living space [1,2]. The results of these experiments, mainly the latter, became the seminal point of many discussions concentrating on the influence of factors such as habitat area and resource availability on the functioning and potential development of populations.

In the most famous experiment [2], the author prepared a closed physical universe as a square with an edge length of 2.57 m. High walls around this pool prevented migration. Mice could not climb to the top of the wall. However, they could reach a particular line, so a part of the walls could be considered to expand the space available for mice. In this area, 15 nesting boxes were mounted. The walls formed 16 cells, each of them containing food suppliers. The system had been thought out so that food and drink suppliers could safely serve about 9500 (food) or 6150 (water) individuals. As will be seen, the population never reached this size. The initial set of 8 mice was chosen so that no disease of epidemic character could be expected. The author also eliminated all risk factors, like predators, from the environment.

**Figure 1:**
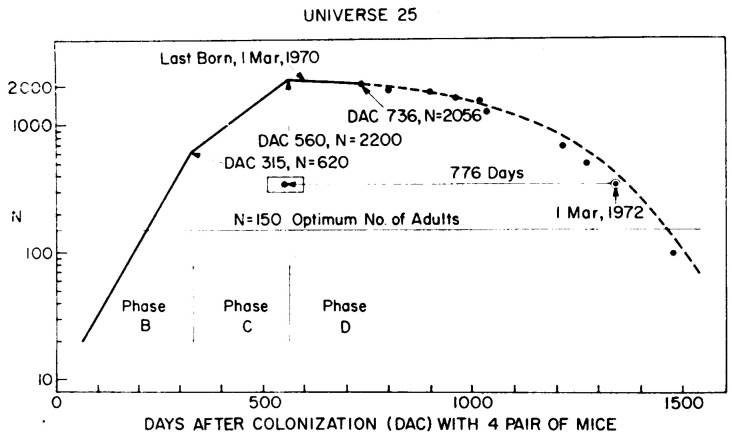
The original plot from Calhoun’s paper. The line presents the approximation of the development of mice population in a closed environment.

The history of the population is shown in the original image from Calhoun’s paper; see Figure 1.

The crucial observation on this famous plot is that after initial growth, the population started to decline. The main point of the discussion was the reason for this community fall. Even in the discussion that followed the publication of the paper, debaters raised different objections concerning the possible sources of the problems. There were, e.g., failure to maintain sanitary conditions, the color coding of mice and the influence of this artificial marking, external factors influencing the lack of symmetry of litters’ birthplaces, or the relation between the state of mice and the hormone levels. Some debaters also discussed the problem, which is still valid today. Did the problem come simply from overcrowding or some other associated factor? Comparing the density of highly populated cities in the world (Hong Kong was then an example) and the densities during the experiment, the author showed that additional effects have to be considered. He pointed to abnormal behavior, which is connected but not entirely explained by the high density.

Interestingly, Calhoun’s paper inspired many studies that either directly correspond to the ideas of the experiments or try to enhance the results and conclusions on human societies. As an example, we can show the paper by Kovac [3], which extends conclusions to the contemporary society, pointing to “hyperconsumerism, hedonic treadmill, disintegration of spiritual foundations of society, post-truth machination” as factors leading to social collapse by increasing the number of sensory signals. A similar thread was studied in the work in [4] as a work being inspired by and simultaneously being the inspiration for the many social fears of those times. The behavior of some individuals that aim to exert dominance over others has become the basis for optimization metaheuristics [5]. The neurobiological effects of social stress have been studied, e.g., in [6], where authors visibly refer to Calhoun as a pioneer of such studies. An exciting area that is mentioned as a consequence of Calhoun’s study is proxemics. Since then, the study of how humans use their space has become an important problem, studied in the context of many processes; see [7,8].

It seems strange that taking into account its importance and still a certain lack of understanding of Calhoun’s experiment, we were unable to find any approach trying to model it. Sometimes, different agent-based approaches are mentioned in the context of such social phenomena. The crucial problem, however, is that the majority of such studies are conducted in a much better-structured environment or with more grounded, well-defined parameters. These features enable either numerical modeling or advanced analytical analyses, often within the framework of game theory or Reinforcement Learning techniques. As some exemplary propositions, we can enlist here the models where some reward (or regret) is the factor steering the process [9,10]. Unfortunately, we are unable to extract and clearly show the factor that would be responsible for directing the process. We have to base our analysis on behavioral elements that define agents’ behavior. On the other hand, some models use the concept of a leader [11]. This idea cannot be rejected a priori, but in the original experiment, such leaders were not observed; thus, we cannot include it in our model. A similar idea, strongly supported by the consensus technique, as shown, e.g., in [12], can be commented on similarly: we do not have any knowledge about reaching consensus or its mechanism in Calhoun’s paper.

In this paper, we propose general parameters and their values that could lead to the reconstruction of the experiment.

## 2. Method and Results

The simulation is divided into several phases. Initially, we divided it into three phases, corresponding to the ones proposed by Calhoun. In this initial plan, they were supposed to have different meanings in the simulation. However, we finally decided to consider just two phases, joining together phases C and D of the experiment.

### 2.1. Mouse Time of Life

**Figure 2:**
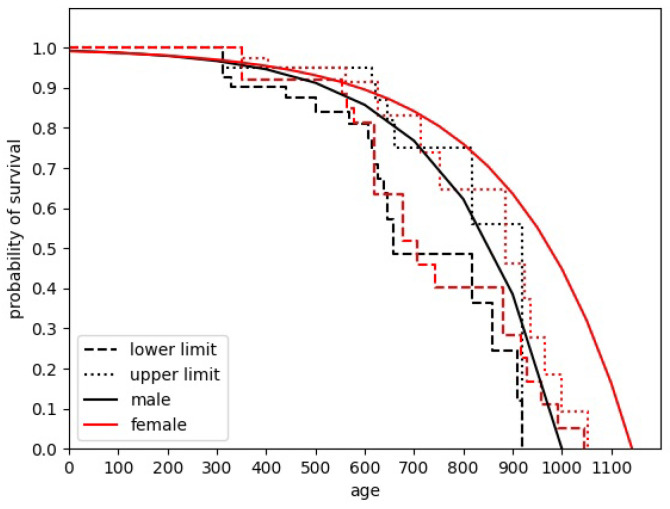
The plot showing the prediction of the length of mice’ life. The dashed and dotted lines correspond to the data from reference [13]. The solid lines are our approximations. The color of particular line corresponds to the gender of mice.

Determining the average lifetime of mice/agents is crucial to correctly estimating population development. Several sources give different, sometimes even conflicting information when trying to analyze this parameter. The approach used across this paper is based on the study by Adelöf et al. [13]. Studying the aging process of C57BL/6NxBALB/c F2 hybrid mouse species, the authors obtained a plot showing the ranges of survival probabilities for particular ages. We approximate it as follows: Firstly, we decide to describe dependence by the exponent-like function. Then, in order to determine the best fit, the “edge” points of the plot are subject to approximation. Using edge points allows us to determine the point where the percentage of surviving mice changes its value. In the original study, also included here (Figure 2), it is seen as the change in the range acceptable for the surviving mouse percentage value.

Finally, we approximate the mean-lifetime curve by the dependence given by $prob_{i,life}\left(t\right)=1-exp\left(-\alpha_{i}\left(T_{i,max}-t\right)\right)$, where *i* corresponds to the sex of the mouse. The problem comes, however, when comparing the parameters with the data given by Calhoun [2]. In the original paper, different species’ populations were reared, particularly Balb C albino house mice. The differences between the data are clearly visible. While Adelöf et al. estimated the maximum lifetime of the male mice as a little bit more than 900 days, while for females, it was about 1050 days, in the final population of Calhoun’s experiment, there were 23 females and 4 males, and no one was reported to be younger than 987 days.

We also decided, a bit artificially, to expand the proposed distribution, enabling its male components to survive for almost 1000 days. This was done by increasing the $T_{male,max}$ parameter from the initial $T_{male,max}=920$ to $T_{male,max}=1000$, preserving the $\alpha_{male}$ parameter. In order to be consistent with observations, we increased proportionally the original $T_{female,max}=1050$ value to 1143. The final form of dependence is shown in Figure 2.

In our opinion, the procedure where the characteristic function is kept with its parameters changed in order describe the real behavior of species used in the experiment is methodologically correct.

The parameters used in the approximation shown in Figure 2 are as follows: $T_{male,max}=1000,\;t_{female,max}=1143$, $\alpha_{male}=4.87\times 10^{-3},\;\alpha_{female}=4.16\times 10^{-3}$.

### 2.2. Phase B—Model Tuning

As mentioned earlier, the so-called phase B is the phase of dynamic population growth. It presents an opportunity to adjust the fundamental parameters related to the unrestricted process of animal herd development. We assume, following Calhoun, that no factor negatively influencing either the behavior of each particular individual or the social relations exists.

In order to study this phase, we have to define the set of parameters. We propose the following set:

- Mate readiness (females).
- Distribution of litter size.
- Probability of male born.
- Role of sector distribution.
- Initial delay.

The significant fact is that we consider the values assigned to all of the above parameters as constant throughout the whole simulation. In the following sections, we will define the other parameters which will modify them.

With mate readiness, we refer to the factor influencing the readiness of females to become pregnant. It is well known that in every population, there exists a number of individuals who do not have the tendency to enter into relationships. A good example is the famous factor 2.1, which corresponds to the fertility rate needed to reproduce the human population. On the other hand, we cannot consider the state of every mouse female as permanent pregnancy. Therefore, the values of mate readiness cannot be too high. We assume that such a factor should correspond to every female mouse in the population and, since the particular probabilities in the situation occurring with increasing population size will be defined elsewhere, it should be, on average, constant for the whole simulation time. Finally, the values between 0.001 and 0.1 were tested.

We can find many approaches when considering litter size, starting even almost 100 years ago. For example, in Watt’s paper [14], the average over 431 litters was reported as equal to 6.12, with a good approximation of the distribution based on the Gaussian distribution and a standard deviation equal to 2.32. It is interesting to mention the above paper since the author observed the phenomenon of cannibalism related to overcrowding cages. Unfortunately, it is impossible to find such information as the species of mice or the aerial density of the population (or the size of cages) for the described experiment. Indeed, more contemporary studies also address this problem, emphasizing, however, different problems. In the paper by Valiquette et al. [15], some neurological properties are studied against the features of particular C57bl/6J mouse litters. Litter-size-related data are not the area of investigation, but some presented data show that the majority of litters are about 6–7 pups in size. The paper in [16] shows clearly the asymmetric distribution of litters of the same C57bl/6J mouse species. There, the maximum is about 9–10 pups. However, litters with even one or two pups exist, but the litter size never exceeds 13.

Following the above data, we made three assumptions concerning litter size. The distribution of litter sizes is a Gaussian distribution; the mean value of the distribution has to be somewhere between 6 and 12, and the standard deviation is in the interval [0.5,4]. Certainly, narrower distributions correspond to better-defined litter sizes, like in [15], and more scattered ones to distributions such as those shown in [14,16].

When considering the percentage composition according to the sex of pups, we can also find a lot of different data. Indeed, mainstream studies are related to the search for the influence of different external factors, but we can try to deduce some more general properties. Among the references available, we can mention, e.g., research on the influence of diet [17], some anatomical elements [18], or even the meteorological situation [19]. Generally, we can expect a slight majority of males in a litter. Therefore, we decided to assume the percentage of males (so-called in the literature the sex ratio) as slightly exceeding 0.5.

A significant problem is the distribution of mice into sectors. Calhoun mentioned visible differences related to the animals born between the 86th and 338th day. The number of newly born mice varied from 13 to 111 in one of the fourteen zones and was not correlated with the zone’s location or the location of food hoppers. The average number of pups was about 60 per zone (the total number was 342), so the extremal values differed very strongly from this average. There are some possibilities to simulate the sector effect. Indeed, the first approach is the most simple one. We neglect the problem and do not deal with sector assignment. On the other hand, the significant differences in the number of births show that it should be somehow taken into account. It is certainly easy to assign the newly born mouse to the zone being the place of its birth. Such an approach can also be studied in our model. It causes, however, some inaccuracies and questions. First of all, we do not know anything about animal mobility. The whole cage is relatively small (a square with an edge of 2.57 m), and emigrations between zones definitely occur. We cannot predict the direction of such processes. As a great danger associated with the assumption of constant residence, we also have to mention the drastically increasing inbred coefficient.

In order to explain the choice of other parameters, we have to briefly describe the course of phase B. Initially (day zero), eight mice, four females and four males, were introduced into the experimental area. Calhoun describes it as a phase of rapid population growth characterized by “5 doublings (generations)” in phase B. This growth led to 620 individuals, of which 150 were adults, on the 315th day of the experiment.

However, some other decisions on our simulation parameters and their choices depend on information often missed in the description of the above experiment, such as information about the first litter born. As mentioned in the paper, it took place only on the 104th day of the experiment. This fact should be confronted with the data related to mouse pregnancy time, usually reported as belonging to the interval [19, 23]. In order to obtain results consistent with both data, we have to assume an initial delay in the mating process. These assumptions are indeed in agreement with Calhoun’s words about the “initial turmoil and adjustment”. We also have to decrease the mate readiness parameter when trying to minimize the delay. Moreover, this change would make it impossible to reproduce further rapid growth. Therefore, we assumed delay values from the interval [60, 90] days.

**Figure 3:**
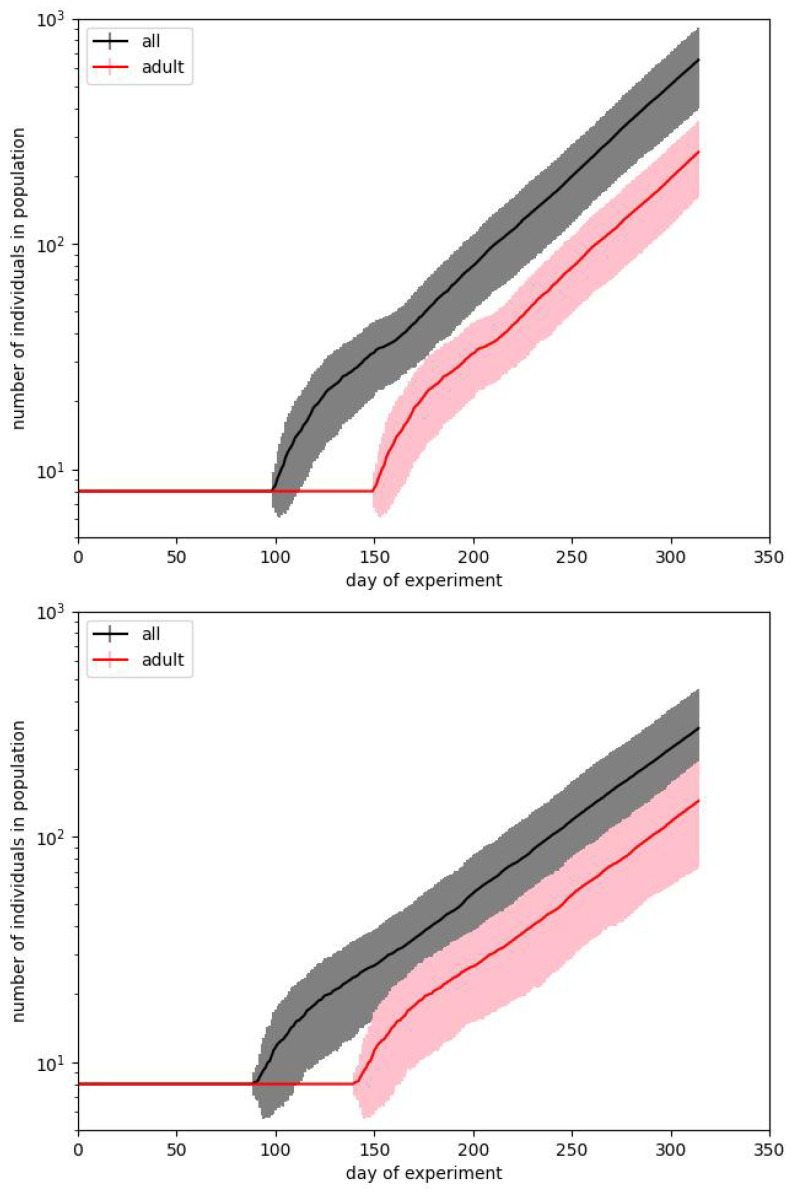
Two simulations best fitting the experimental data during phase B of Calhoun’s experiment. Plots show the courses of the total size of the population and the number of adults. Bars correspond to the standard deviations calculated over 100 simulation runs performed with the same input parameters.

**Figure 4:**
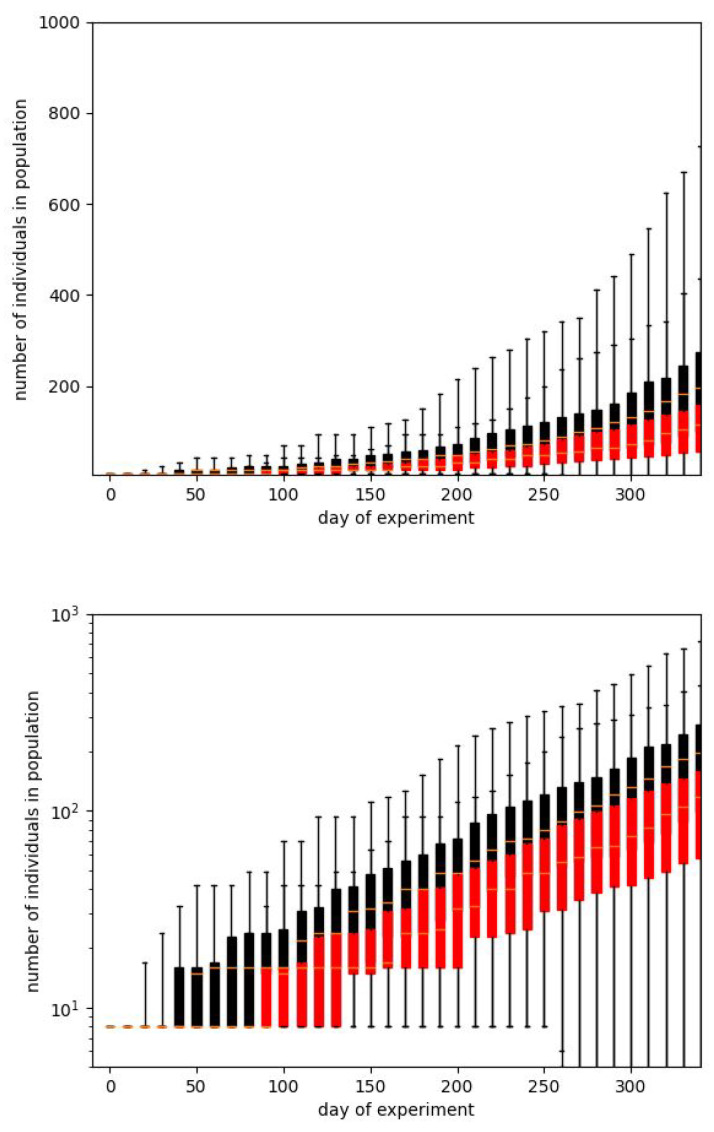
The plots from Figure 3 shown as box plots. This presentation form is common for such a type of statisticaldata. Whiskers correspond to the minimum and maximum values, boxes to the position of the first and third quartiles, and a short line to the median. The plots confirm a large dispersion of results for a particular timestamp and the illegibility of this form of data presentation. As it has been earlier, black color corresponds to the whole population while the red one - only to the adult individuals.

In Figure 3 and Figure 4 we show the same results in two forms. We usually prefer the box plot, since it shows the distribution of values in more detail. However, these plots are hard to interpret, mainly because in some situations the population is dying out. This observation also confirms the suggestion that Calhoun’s result may be a very particular run.

The above comments also give some hints concerning the mate readiness parameter. By clarifying some of Calhoun’s data concerning phase B, we have to notice that indeed, the population grew not by five times but by more than six times. Considering the time between the first litter and the end of the phase, we can establish the time of doubling as about 33 days, which corresponds to about 150% of pregnancy time. This means that about one in four females should go through pregnancy in 33 days, which gives an estimation of mate readiness of about $10^{-2}$.

When starting the simulation, we find that results are very strongly spread on the population scale. This effect can be observed in Figure 3.

The plots show two cases that present the average results close to the values presented by Calhoun for phase B, but they need some comments. The first question is how we have to reproduce the original result. Calhoun’s experiment was performed just once, and we must consider it a particular case. We cannot predict that its reproduction would produce precisely the same result. The majority of social experiments are usually less resource- and time-consuming and can be reproduced at least several times. Such is not valid in the studied case. Therefore, the crucial question is whether, while aiming to reach the same result, we have to fit the average for a particular parameter set or find the single runs which are the closest to the benchmark results for the given days of the experiment.

Figure 3 corresponds to the first case. For every set of parameters, we make 100 simulation runs and average values for particular times. The length of the bars corresponds to the standard deviations obtained for these runs. We show the curves for the total number of individuals in a population and for the number of adults. Such a way of presentation is related to the fact that it is reported that on the 315th day, the whole population counted 620 mice, but there were just 150 adults among them. It turns out that although it is straightforward to reproduce the exponential dependence of one of these numbers, it is pretty hard to reproduce both of them.

Some of the set of parameters leading to the results being consistent, on average, with the experimental ones are shown in Table 1.

In Table 1, we show some sets that can produce the best results when fitting to the original course of the experiment. We obtained the “best” solutions by analyzing simple values:

(1) $$consistency=(\frac{n_{total}-640}{640}{)}^{2}+(\frac{n_{adult}-150}{150}{)}^{2},$$

So, indeed, we are looking for the minimum sum of relative squares for the total number of individuals ($n_{total}$) and adults ($n_{adult}$) compared with the expected values of 640 and 150, respectively.

There are several factors that stand out here. First of all, there are a lot of sets of parameters leading to similar results. Table 1 contains some chosen arbitrary sets to show a relatively broad spectrum of parameter values, enabling a similar final effect. It seems to be in some contrast with the earlier note about the difficulty of obtaining correct results. However, we must note that the relative values corresponding to the minimization of the metric in Equation 1 reach a deviation of not less than about 10% from the expected ones.

Additionally, we have to emphasize some simplification in the figure shown in the original paper by Calhoun. The line corresponding to phase B is drawn, starting from the point (0,8). It is certainly the effect of extrapolation. Taking into account that the first litter appeared on the 104th day, the line should be much steeper.

Coming back to Figure 3, we notice that usually, after about 50–80 days, after some characteristic point defining the departure from the starting level, the characteristics are of pure exponential type. The important observation is, however, that there are very large deviations from the average results. The presented plots, drawn in the lin–log scale, show that the standard deviation arises with time and reaches several tens of percent of the average value.

**Figure 5:**
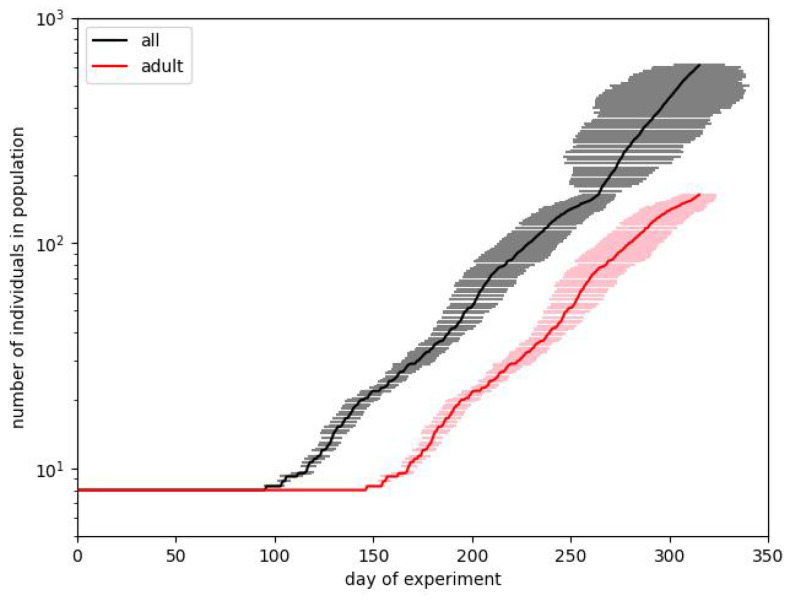
The modeling of phase B is presented differently. The horizontal whiskers correspond to the standard deviation on the day when a particular population size was reached.

In order to somehow study the single runs that lead to the best results, we selected those runs that were relatively close to the expected one. The choice is to save those runs for which the total number and the number of adults at the end of phase B differ by an amount not greater than 20 from the expected ones. We should mention here that we obtained only tens of acceptable results after performing thousands of runs. The smallest confidence metric value (see Equation (1)) was about $2\times 10^{-3}$, corresponding to the pair of final numbers (629,156), so indeed, we never hit the perfectly correct pair. In Figure 5, we show the data for such selected runs. The error bars, however, relate to the number of days when the given result was obtained. Here, we can also observe the increasing standard deviation, but this increase is not monotonic like in Figure 3.

The above comments show clearly that we have to assume that the data provided in Calhoun’s experiment described a particular run and cannot possibly be considered to be explained by some set of parameters. Instead, we have to take into account the broader intervals of particular parameters and look for expected behavior in single runs. When trying to summarize some conclusions about the selection of parameters, we can show the possible areas. Certainly, among the parameters, we have the ones related to biological factors. Among them, we can enlist, e.g., the size of litter distribution. Here, we can say that we obtained the expected results since we obtained the best fit for Gaussian distributions with a mean of about 8–10. An interesting observation is that acceptable results can be obtained for the wide (σ=4), as well as for very narrow distributions. σ=0.5 (not listed in Table 1 but also present in the simulations) means that we obtain values different from the mean with a minimal probability. Also, the sex ratio for litters confirms the knowledge about the slight (and only slight) overrepresentation of males in litters.

The other parameters try to cover some possible areas of search. For example, the distribution over sectors turns out to be utterly useless if the simplicity of its inclusion is too far. The time delay, which can seem a little bit artificial but corresponds to Calhoun’s “turmoil,” led to expected values of about 70. We assign the crucial role in the simulation, however, to mate readiness. Also, here, we can say that the values corresponding to the best results confirm some preliminary expectations.

Considering subsequent phases, there is a need to find the processes that should lead to a particular behavior (e.g., growth slowdown and final decrease) rather than to fit to the concrete curve.

### 2.3. Phases C and D—The Decline in the Population

In our initial plan, we wanted to study phases C and D separately. The main reason for this assumption was that phase C can be considered a slowing of the development process. Also, the presentation of these phases in Calhoun’s original plot suggests that something happened, but the population was in slow but continuous development. Initially, we wanted to manipulate the simulation slightly with the parameters; however, ultimately, we decided that both phases represent the process of deepening population destruction. The idea was to observe the beginning of problems and the possible factors that can slow down the growth of the population (phase C) and then to observe if the intensification of these processes can lead to the destruction of the society. It turns out, however, that we must consider both phases together. The attempt to find the maximum position of the population plot needs to be studied from the perspective of the whole pathological process.

It is important that we retain the parameter values used so far. This step may be disputable, since changes to any parameter in Table 1 can influence the process. Mate readiness has a social background and is directly reflected in the number of newborn individuals. The same can be said about litter size; here, we can suppose biological reasons. We think, however, that including such modifications may obscure the influence of the elements identified by Calhoun.

In order to include the destructive processes, we have to follow the original observations by Calhoun, who indicated the following social factors characterizing the mice’s behavior and, finally, indicating the negative processes:

1. The lack of social niches, with individuals who did not find their roles in the community becoming inactive. Additionally, they started to present non-territorial behavior by occupying some selected places, usually in the center of the pool (male-related).
2. The increase in the level of aggression. This process especially concerns territorial individuals, attacking the withdrawn ones (male-related).
3. The increase in the level of aggression. A similar point to the above also concerns females. This process is especially true when it concerns nursing mothers. It is presented as taking over territorial male roles by mothers (female-related).
4. The disruption of maternal care. Two processes were observed. The first one is the total abandonment of litter, while the second one is the lack of care for individual pups when, e.g., moving into another nest. Finally, it causes the death of either all litters or some newly born mice (female-related).

We try to implement the above deviations by including into the simulation scheme several parameters. The parameters mentioned earlier, which were defined to describe the “unlimited” process in phase B, do not change. They are only modified by the values listed here:

- Critical density for individual processes.
- Critical density for social processes.
- Probability of litter death.
- Individual deviation factor.
- Probability of belonging to the group of “beautiful ones”.

Both densities can be described together. These numbers can obviously be defined in the absolute way. We know the size of the pool (2.57×2.57 $m^{2}$), and we can refer to the number of individuals living in such area. Due to the constancy of the above size and simplicity of presentation and calculation, we decided to use just the number of mice as a critical value. Its influence on simulation runs will be presented after some comments on the other parameters.

Probability of litter death is related to the fourth point of the above list of deviation factors. Two processes are described at this point, and by including the described parameter, we want to refer only to abandonment. After crossing the threshold given by critical density for social processes, with some predefined probability, the mother leaves her litter unattended. In the simulation, this means that (1) the mother is excluded from the possibility of getting pregnant for the time of the pregnancy that successfully ends with delivery and (2) the whole litter is not added to the population.

The “beautiful ones”: the concept was used by Calhoun for the description of males who did not engage in fighting and sexual contacts. They are distinguishable from the other males, either territorial or non-territorial, since they do not have any wounds. We assume that also after crossing the critical density for social processes, some percentage of males given by the separate parameter become “beautiful ones”, and this state is permanent until the death of the individual.

The individual deviation factor seems to be the most important when studying the processes of destruction of the population. The name can be a little bit confusing, since a more abnormal behavior corresponds to lower values of the parameter and not, as it can be directly deduced from the name, to higher ones. This factor means something different for males and females but always belongs to the interval [0, 1]. For males it is used when a possible pregnancy is analyzed. When the potential father is selected from the list of males, the individual deviation factor determines the probability with which the chosen individual really becomes the father. Thus, the factor reduces the success of becoming pregnant for the female in contact with the selected male. For females, we assume that this factor corresponds to the number of pups in the litter. The number sampled according to the rules presented earlier is multiplied by the individual deviation factor, thereby decreasing the litter size. The value of individual deviation is determined in such a way that initially, all members of population have it set to 1. When going beyond the critical density for individual processes, the value is multiplied by the inverse of the quotient of population size and critical density. This means that the value never increases for a particular individual. Since every individual in the population is subject to this procedure, the average value of this parameter also permanently decreases. For newborns the value is set as the average of the parameter known for the mother and father. So this procedure can produce individuals with a parameter higher than that of one of the parents, but at the same time, this cannot significantly rise the average value for the population. We hope that such a definition can keep the parameter at a higher level for longer, since generally mice with a higher level of individual deviation take part in reproduction.

**Figure 6:**
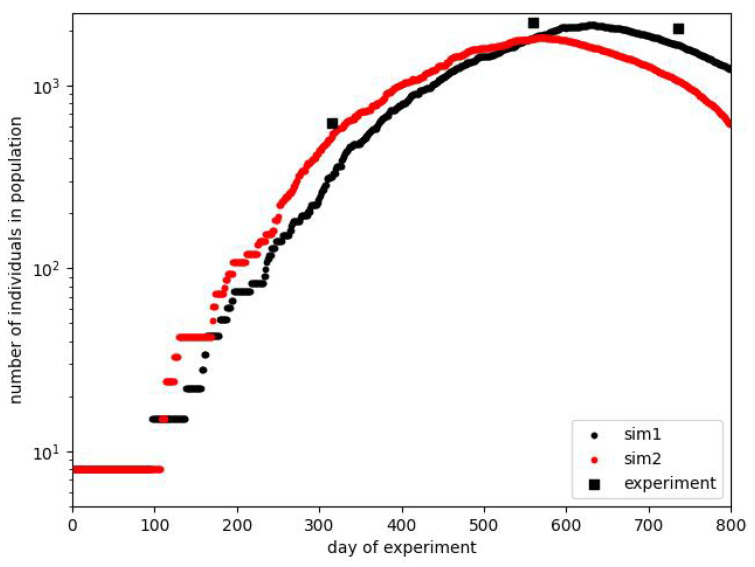
Plots of exemplary simulations showing the existence of the effects taking place in Calhoun’s experiment. For comparison, the data measured in the experiment are shown in the form of discrete measurements. The real experimental data, not burdened with the authors’ prediction or approximation, contain only such points.

Finally, we can show the course of some simulations, which can be considered, in general, to follow the original plot. We present them in Figure 6. The figure contains only the plot for the total number of individuals since we do not know the exact relation between the number of adults and pups at arbitrary moments of the experiment. Indeed, we know it only at the end of phase B, as it was considered earlier. For the final stage, exceeding the period shown in the figure, we know only that there were no births after the 600th day; it is reported that after the beginning of the experiment on 9 July 1968, the last litter was born on 1 March 1970. All other data concern the whole population.

As mentioned earlier, the course of the experiment was a particular one. It took much work to reproduce it with very high accuracy. In Figure 6, we show two courses close to the experimental one. Intentionally, we do not show the results after the 800th day because different manipulations, like the removal of part of the population, took place during this time. The crucial thing is that we can reproduce the approximate shape of the population curve. The maximum position is approximately correct, but we are unsure whether the 2200 individuals on the 560th day is the exact measurement or some estimation. The measurements of population size were not performed every day. The effect that can be under more detailed consideration is the width of the maximum plateau, which can be extended more.

However, we think that the presented results confirm the selection and definition of parameters with a need for attention to be paid to their better tuning.

## 3. Conclusions

In this paper, we present the results of the simulation of Calhoun’s experiment, usually called “mouse utopia,” where a mouse population, after initial dynamic growth, is finally extinct. This effect happened despite the lack of any life-related problems. According to the author’s interpretation, the reasons for such a process were mental problems related mainly to the overcrowded environment, which turned off the emigration process.

We must again remind the reader that the animals were fully supported with food and water, and we also know the detailed distribution of food hoppers and waterers. However, the most significant problem with simulations like the one presented is that we have problems connecting individuals’ spatial and mental behaviors. We can try to describe the sources of mouse behavior, but by trying to simulate the experiment in detail, we would observe difficulties. The primary source of these problems is the fact that every single process should be described, implemented, and monitored. A good example here is the study of aggression directed to non-territorial males. We do not have detailed knowledge about the events being the cause of wounds. Were they attacked, e.g., trying to achieve a hopper with food, or was the attack completely unprovoked? The simulation of such effects also requires a change in the time step length. In our model we can simply use one day as one time step, but when reproducing detailed behavior, we have to shorten this time. The shortening to one minute would be, in our opinion, sufficient to enable the time step to cover particular events.

The next element that makes the simulation harder is that we do not know many details about the experiment. Calhoun was an ethologist, and his work concentrated on the description of behavioral processes. He did not present many detailed data, which could be helpful for computational modeling.

We must emphasize that despite the lack of much information, we correctly reconstructed the qualitative course of the experiment and are pretty close to doing it also quantitatively. Moreover, we can ask some questions concerning Calhoun’s theses. For example, let us show his note in the original, referenced here in Figure 1. He wrote that the optimal number of adults is about 150. We are not quite sure what “optimal” means. However, in the simulation, the critical densities leading to the best approximation were about 300–400, so by preserving the ratio between the total size of the population and the number of adults at the end of phase B, the number of adults should be about 75–100.

When presenting the obtained results, we must return to one of the basic questions concerning them. To what extent is Calhoun’s experimental result unique, and to what extent can we consider it repeatable? Studies usually conducted on human societies [20,21,22] confirm the obvious fact that the stochastic behavior of individuals corresponds to and influences the stochastic behavior of communities. Therefore, we can conclude from the presented result that the model corresponds to the general character of a population guided by certain, statistically interpretable parameters and that among the possible solutions, the course observed by Calhoun is completely acceptable.

We can certainly show the most interesting further direction. In our opinion, it should especially contain two issues: the individualization of parameters and making them time-dependent. We must also express the main objection here that we cannot find any methodological basis justification to introduce any models. We hope, however, that we can try to study in more detail the processes that turned out to be hard to describe (e.g., total/adult ratio and maximum peak).

In Calhoun’s experiment, the mouse population disappeared after a few years of experiment after initial successful growth. Different processes were blamed for this effect, from overcrowding to excessive prosperity. We want to avoid joining this discussion. We can certainly indicate the factors that are more important for the detailed result than others. We can, e.g., almost completely neglect the distribution of new litters among zones, while the shape and parameters of litter size play a more significant role. From Table 1, we can conclude that the distribution of litter sizes that best fits the results is the Gaussian distribution, with the average changing by 20%. On the other hand, a very important factor is the initial delay in adapting to environmental conditions. The results presented in the paper show that we can simulate the effect, but although the parameters were mainly connected to the spatial density of the population (following Calhoun’s remark “However, in my experimental universe there was no opportunity for emigration.”), some other mechanisms can be proposed to explain the changes in these parameters.

## Figures and Tables

**Table 1:** The exemplary results of simulation parameters leading to the results consistent with experiment.

	Set 1	Set 2	Set 3
mate readiness	0.01	0.01	0.02
litter distribution type	Gaussian	Gaussian	Gaussian
average/min	10	8	10
standard deviation/max	1	2	4
probability of male born	0.54	0.52	0.52
role of sector distribution	none	none	none
deviation of sector	n/a	n/a	n/a
time delay	60	60	70

## Data Availability

The raw data can be accessed: https://github.com/tomgwizd/Mouse-utopia-model/blob/main/Mouse-utopia_data.zip, accessed on 7 January 2026.
